# The relationship between nutrition literacy and nutrition information seeking attitudes and healthy eating patterns among a group of palestinians

**DOI:** 10.1186/s12889-023-15121-z

**Published:** 2023-01-24

**Authors:** Mariam Al Tell, Nihal Natour, Eman Alshawish, Manal Badrasawi

**Affiliations:** 1 Department of Public Health, School of Medicine, An-Najah National University, Nablus, West Bank, Palestine; 2 School of Nursing, An-Najah National University, Nablus, West Bank, Palestine; 3 Department of Nutrition and Food technology, Faculty of Agriculture and Veterinary Medicine, An-Najah National University, PO. Box 7, Tulkarm, West Bank Palestine

**Keywords:** Nutrition literacy, Diet, Information, Behavior

## Abstract

**Introduction:**

Nutrition literacy is crucial because it gives people information and drives them to take responsibility for their eating habits. Prior research on three categories of nutrition literacy among Palestinians was lacking: functional literacy (FNL), interactive literacy (INL), and critical literacy (CNL).

**Aims:**

(1) Describe three types of nutrition literacy—FNL, INL, and CNL—among a group of Palestinians was one of the study’s primary objectives. (2) To investigate the connections between various nutrition literacy levels, eating habits, and the habit of seeking out nutrition-related information.

**Methods:**

149 Palestinians were chosen at random to take part in the study in the fall of 2020. Data on sociodemographic variables were gathered through an online survey that was disseminated across social and educational internet sites. Nutrition literacy data was gathered using a translated questionnaire, while diet behavior data was gathered using the Short Format of the Diet Health and Knowledge Survey (SFDHKS). The data were examined using SPSS 21.

**Results:**

This study included young people (20.4 ± 4.9 years old), 78% of whom were female. The majorities of participants had bachelor’s degrees or were already enrolled in school to obtain them. FNL had a mean of 2.8 ± 0.5, INL of 3.3 ± 0.5, and CNL of 3.6 ± 0.5. The connection between CNL and INL was significant (p 0.05). Significant correlations were found between many aspects of diet behavior, the usage of food labels, and nutrition literacy.

**Conclusion:**

Participants from the Palestinian community are willing to learn about and comprehend nutrition facts and how it relates to diet behavior in 2021.

## Introduction

Olive oil, green leafy vegetables, grapes, and other fruits and dairy items are all abundant in Mediterranean-style Palestinian cuisine [1]. Similar to other middle-income nations, there is currently a health shift taking place, which has led to the adoption of a westernized lifestyle that is centered on fast food and restaurant meals that is laden with salt, sugar, refined carbohydrates, fried meat, and potatoes [2], [3]. Obesity and chronic illness rates are rising in the Palestinian territory along with changes in dietary practices. Recent statistics indicate that 65.3% of Palestinians are overweight or obese [4]. Currently, the major causes of death in Palestine are diet-related non-communicable diseases, such as cardiovascular diseases, stroke, cancer, and type 2 diabetes [5]. It is common knowledge that poor food choices and inactivity are major risk factors for chronic disease death [6].

The rise in noncommunicable diseases in Palestine needs to be stopped, and efforts should focus on dietary choices and other behavioral factors. Factors that may affect food choices include personal, interpersonal, social, and cultural [7]. The key component in preventing diet-related chronic diseases in any population is nutritional awareness, which includes the capacity to comprehend and apply nutritional knowledge. The capacity to access, absorb, comprehend, and apply health knowledge and practices are what is meant by the concept of health literacy [8]. In addition, because they connect consumers with the rapidly evolving food environment, concepts like nutrition and food literacy are important for human health [9]. 1992 saw the introduction of food literacy in cookbooks [10]. Functional nutrition literacy (FNL), interactive nutrition literacy (INL), and critical nutritional literacy (CNL) are the three types of nutrition literacy that Nutbeam’s tripartite model has recently found [11, 12].

FNL refers to consumers’ basic skills and abilities to obtain, comprehend, and apply nutrition information. INL refers to a consumer’s ability to participate in the communication of nutrition information, to share and discuss it. Finally, CNL refers to consumers’ ability to evaluate and critique nutrition information, as well as understand the relationship between food and the environment [13]. The primary significance of nutrition literacy is its impact on eating habits [14]. Although the literature on the relationship between nutrition literacy, diet behavior, and BMI was inconsistent [15], and some reports strongly support this relationship in children aged 10 to 12 years [16], adult studies are needed.

It is well known that a high intake of whole grains, vegetables, fruits, nuts, and fish is associated with a decrease in all-cause mortality, whereas a high intake of groups such as red meat, processed meat, and refined grains is associated with an increase in all-cause mortality [17]. In the United States, studies show a strong link between poor nutrition literacy and poor nutrition knowledge and practices, the development of chronic diseases, increased hospitalization, and cost [18].

Palestinian society must play a role in raising global awareness about the importance of diet in disease prevention and improving longevity and quality of life. This is better reflected in the level of nutrition literacy, which has not been adequately studied in Palestinian society. Work in this area will help to identify gaps in societal health needs, which may draw the attention of governmental or international funding to improving access to credible nutrition knowledge, which will eventually translate into better practices. As a result, the objectives of this study are as follows: (1) to investigate patterns of FNL, INL, and CNL in Palestinian society. (2) To investigate the relationship between nutrition literacy, food behavior, and label use. (3) Research the barriers to obtaining nutrition information.

## Methods

A cross-sectional design was used to assess nutrition literacy and its relationship with dietary habits. Palestinians over the age of 18 were recruited using an electronic data collection tool that was distributed via various social media platforms, including Facebook and professional, social, and student Facebook groups, as well as the university website. All Palestinians living in the West Bank, Gaza, and Israel made up the population. We used a convenient sampling method and were able to include a sample size of n = 149 adults in this study. This study was primarily representative of young Palestinian students and early-career workers. The inclusion criteria were all Palestinian participants who could use an electronic survey form and read the announcement. Participants under the age of 18 were excluded. We did not exclude participants based on their health status, but the majority of our participants were young.

The data collection tool was chosen after conducting a literature review [19] and the conceptual framework development is described below. Age, weight, height, diet, use of food label, items of food label used (the use of food label was considered a part of healthy habits because we did not use the most recent vital sign evaluation of nutrition literacy, food label was not considered part of nutrition literacy measurement). Gender, education, and income were also collected. Nutrition literacy, including three subtypes, was calculated using a translated questionnaire [19].

Each questions’ answers were given likert scale number and ranged from strongly disagree [1] to strongly agree [5]. The Arabic-translated version of the Diet Health and Knowledge Survey (SFDHKS) [20, 21] was adapted and used to assess nutrition behaviors. It consisted of nineteen questions from the SFDHKS that measured the use of food labels, consumption of low-fat/low-calorie foods, consumption of fiber, and avoidance of extra fat. The author translated the study tools into Arabic and validated them for content validity and feasibility. The translated questionnaires were used in SFDHKS with minor modifications.

**Fig. 1:**
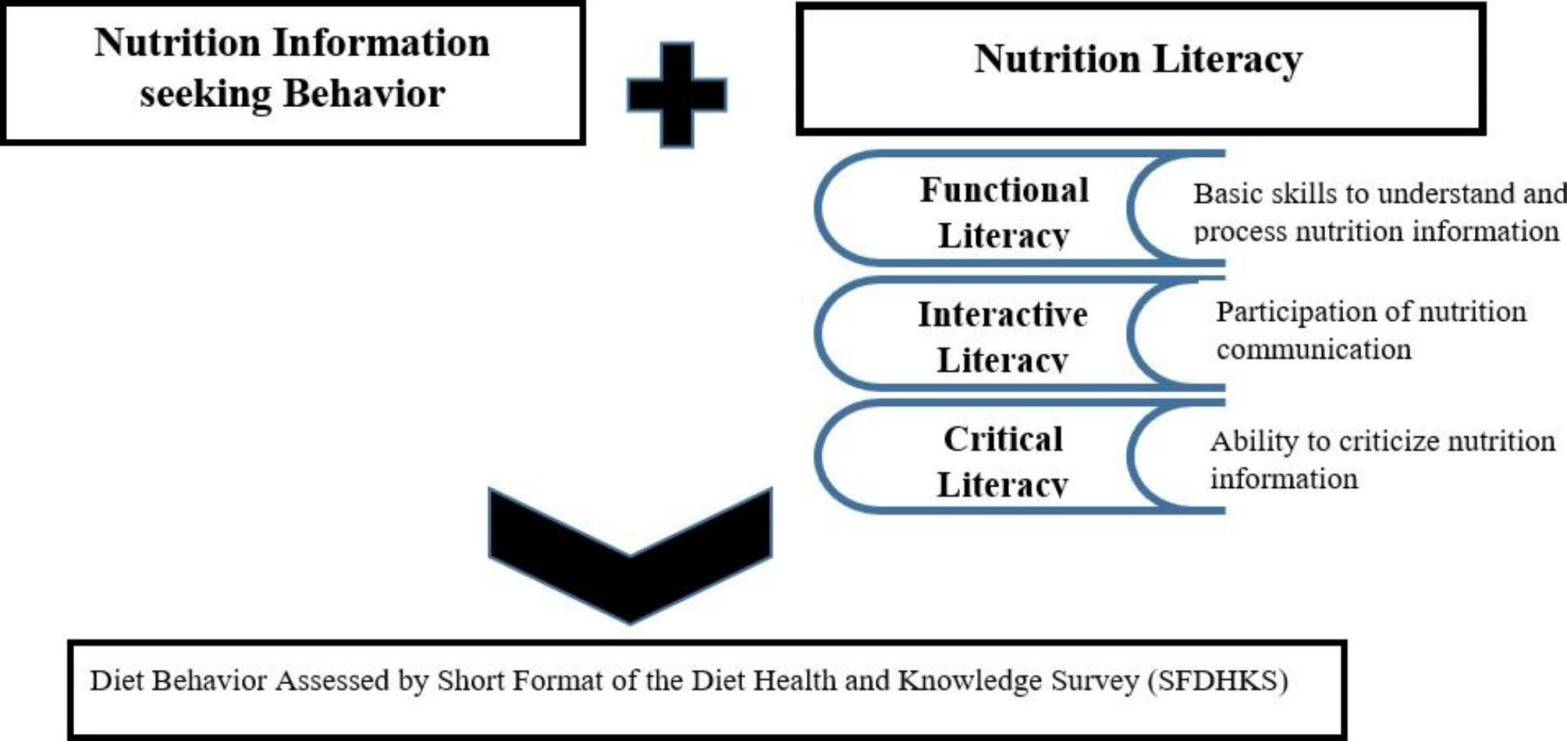
Conceptual Frame work for Study

Figure 1 depicts the conceptual framework for this study, which includes the definition of three types of nutrition literacy (FNL, INL, and CNL). This conceptual frame was developed based on a study that addressed three types of nutrition literacy in Kampala, Uganda, using 29 attitude statements classified as FNL, INL, and CNL [19]. Furthermore, the tools developed in the Kampala Study captured health information-seeking behavior using a study by Zollner et al. 2009 [22]. Questions included confidence in seeking nutrition information or advice, barriers to seeking nutrition information, and level of trust in various sources of nutrition information [19]. The purpose of this study was to investigate the relationship between different structures of nutrition literacy and diet behavior as measured by (SFDHKS) [20, 21].

## Statistics

The study’s demographic variables were summarized using proportions or means. Ordinal data were assigned numbers on a Likert scale ranging from 1 to 5, and it was summarized using means and standard deviation. Normality was checked, and histograms were provided. Pearson correlations were used to assess the relationship between FNL, INL, and CNL and food label use, diet behavior, and nutrition information resource measures. Significant values are less than p 0.05. The data were analyzed with IBM SPSS 21.

## Results

This study included adults in their twenties (20.4 ± 4.9 y) who have a BSc or are currently enrolled in programs leading to a Bsc degree. Our study’s participants are mostly female. The income ranged primarily between 860 and 1719 euros.

In this section, data summarizing the Likert scale meansSD related to FNL, INL, and CNL, as well as the rest of the factors related to seeking nutrition information, are provided (Table 1). In summary, CNL had the highest Likert scale value and FNL had the lowest.

**Table 1:** Description of Study Variables

Study Variable	Percent or means
Age (y)	20.4 ± 4.9 (n = 148)
BMI (Kg/m^2^)	22.2 ± 5.4 (n = 147)
**Gender** Male Female	33 (22%) 118 (78%)
**Work** No Yes	137 (91%) 14 (9%)
**Education** High school or less College Bachelors Graduate studies	11 (7.3%) 19 (12.6%) 117 (77.5%) 4 (2.6%)
**Chronic Disease** No Yes	135 (93.7%) 9 (6.3%)
**Income** Less 860 Euro 860–1719 shekel More than 1719 shekel	49 (33.1%) 54 (36.5%) 45 (30.4%)
FNL	2.8 ± 0.5
INL	3.3 ± 0.5
CNL	3.6 ± 0.5
**Food Label Use** Yes No May be	54 (36.5%) 54 (36.5%) 40 (27%)

FNL, Functional nutrition literacy, INL, interactive nutrition

literacy, CNL, Critical nutrition literacy,BMI, body mass index

### Functional literacy

FNL was made up of seven points. The lowest Likert scale for participants was for their understanding of what a balanced diet consists of, their awareness of WHO recommendations for a healthy diet, and their ability to apply healthy diet principles to their daily diet style. Participants had higher Likert scale scores indicating positive attitudes when it came to understanding information provided by dietitians and using health languages. (Table 2)

**Table 2:** Summary of Various Questions related to FNL, INL and CNL

	Question	Likert Score
Functional Literacy		
1	I find it hard to understand the language used by dietitians, nutritionist and health workers and experts	2.5 ± 0.7 (132)
2	I find it hard to understand the terms and concepts used by dietitians, nutritionist and health workers and experts	2.66 ± 0.9 (134)
3	When I read informationabout nutrition, food and diet plans, I find difficult to understand.	2.15 ± 0.76 (135)
4	I find it difficult to know how to change my diet when I am given advice from doctor or nurse or others	3.33 ± 1.02(136)
5	When I read information about nutrition and food I need somebody to help me to understand them	2.35 ± 0.93 (136)
6	I am not aware of WHO recommendations for intake from fruits and vegetables	3.34 ± 1.02(136)
7	When I read a report on food and nutrition I find words that I can’t understand	2.71 ± 0.88(136)
8	I am aware of the balanced diet concept	3.60 ± 0.88 (136)
**Interactive Literacy**		
1	I collected dietary information from many suitable sources	3.00 ± 1.0 (136)
2	I use internet when I search for dietary information	2.66 ± 0.9 (134)
3	I discuss nutrition matters with my family and friends	3.40 ± 1.06 (136)
4	I change my dietary habits based on my knowledge I gathered from differenct sources	3.04 ± 1.07 (136
5	I do not follow public discussions on nutrition on TV, radio or other media outlets	3.44 ± 1.07 (135
6	I sometimes read material on what represents balanced diet	3.20 ± 0.97 (135)
7	I am ready to take initiative to discuss healthy diet with health professionals	3.52 ± 0.99 (136)
8	When I want nutrition information I do not know which health department I can ask for help	2.93 ± 1.07(133
9	I discussed my nutrition beliefs with others (e.g. friend, family, etc.)	3.42 ± 1.04 (133)
**Critical Literacy**		
1	I want to be able to participate easily in any debate on our food and nutrition system in our country	3.56 ± 1.02 (149)
2	I am ready to take active role in any plan to improve nutrition habits in my school or work place	3.69 ± 0.86 (149)
3	I expect my school or work to offer healthy meals	3.64 ± 0.89 (149)
4	I try to influence others (e.g. family or friends to take care of their nutritional health and habits	3.66 ± 0.92 (149)
5	It is important for me that my school or work place have healthy meals or food	4.32 ± 0.69(148)
6	I tend to be influenced by dietary advice on media outlets such as TV, radio and newspapers	3.56 ± 0.91 (148)
7	I have confidence in dietary plans I read about in newspapers, magazine, and other media types	3.56 ± 0.91(149)
8	I tend to be influenced by dietary advice from family or friends	3.45 ± 0.92
9	I think that publishing scientific evidence on food and nutrition by media is correct	3.39 ± 0.88(149
10	I find it is hard to identify the differences between scientific and un-scientific information on diet and nutrition	3.39 ± 0.88(148)
11	When I read information on nutrition it is important that it is based on scientific evidence	4.27 ± 0.78(147)

### Interactive literacy

The INL is made up of 8 points. Participants scored higher on INL factors than on FNL. INL was created to find nutrition information resources and to share that information with friends and family. The participants appeared to be skeptical of the internet as a source of information. (Table 2)

### Critical literacy

Participants in the study expressed positive attitudes toward engaging in nutrition change by adopting a healthier diet at the social, workplace, family, and friend levels. They expressed a strong desire to have healthy meals served at work, university, and school. They also expressed a desire to persuade others to make healthier dietary choices. (Table 2)

### Barriers to seek nutrition information

Participants in the study disagreed on the difficulty of nutrition information as a barrier to seeking nutrition knowledge on diet and healthy dietary guidelines. However, study participants agreed that the lack of Arabic resources for nutrition information, as well as the credibility of available sources, could be barriers to nutrition information use. (Table 3)

**Table 3:** Barriers to seek nutrition information

	Variable	Likert scale mean ± SD
1	I have to do a lot of efforts to obtain information	3.14 ± 1.06 (147)
2	I can’t make sure of information credibility	3.50 ± 0.97 (147)
3	It is hard to understand nutrition information	2.62 ± 0.90 (146)
4	There is no enough information about nutrition in Arabic, all in English	3.40 ± 0.98 (147)
5	It takes a lot of time to search for information	2.98 ± 0.88

### Relationships between different types of literacy, food label and diet behavior

**Fig. 2:**
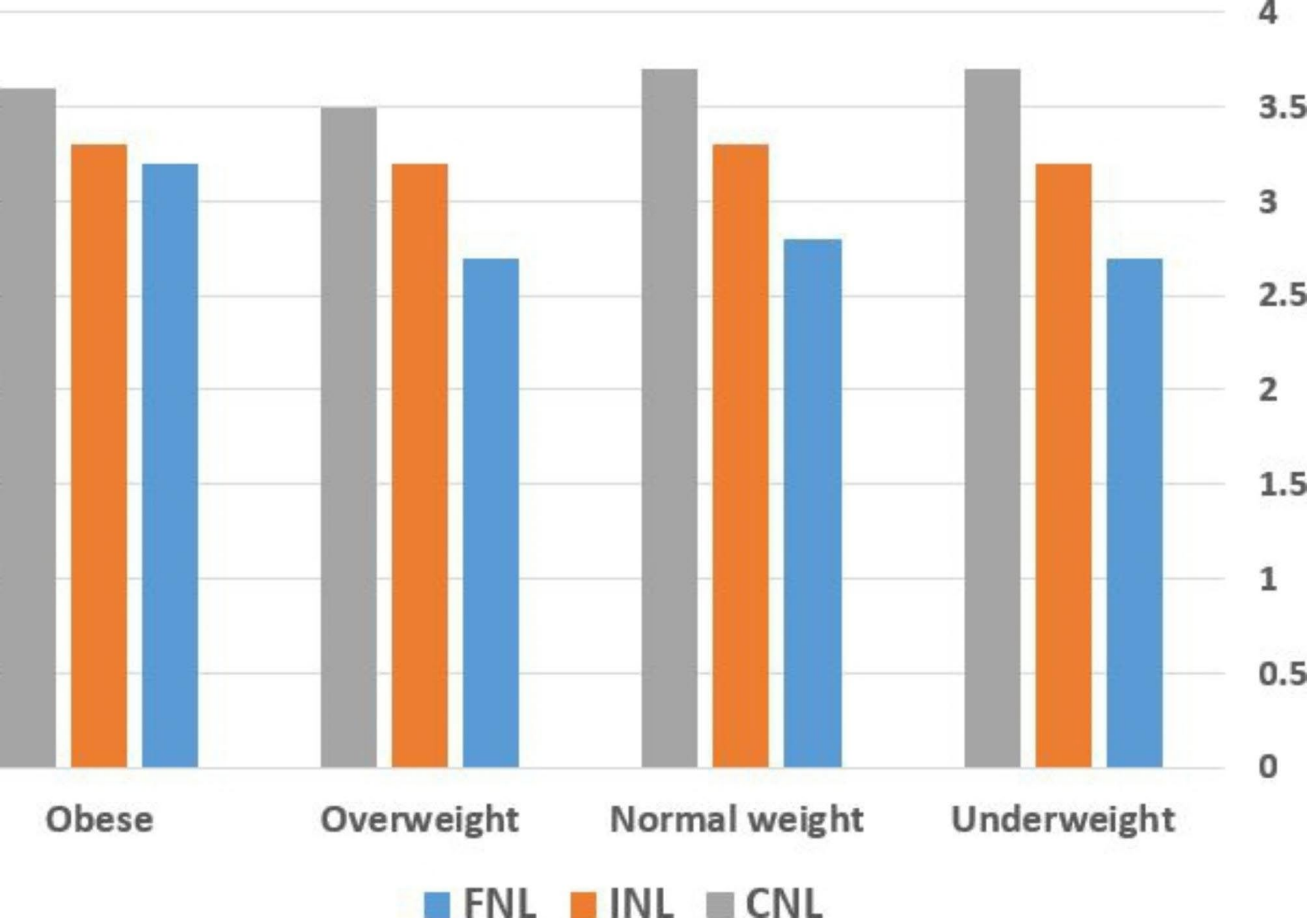
Nutrition Literacy According to Weight Category Measures are based on Likert scale

FNL did not correlate with CNL or INL in this study, but it did correlate with food label use. Looking at food label ingredients and points related to low-fat, low-calorie food, serving size, and claimed health benefits both correlated with CNL and INL. Seeking low-calorie, low-fat products and cooking correlated significantly with INL, whereas adding cheese and mayonnaises was less common in CNL participants. FNL and nutrition information seeking had a significant correlation (Table 4). Participants with better FNL seemed to rely on health professional, scientific books rather than internet. Participants with higher education showed higher average CNL ( 3.52 ± 0.48 versus 3.71 ± 0.45, p = 0.03), whereas there were no differences between FNL and INL according to education. Incomes was not significant predictors of FNL, INL and CNL. (Table 5). Figure 2 provides comparison between three different subtypes of nutrition literacy according to weight category.

**Table 4:** Pearson correlations of Study variables with FNL, INL and CNL and Diet Behavior and Food Label Use

Variable	FNL (131) R^2^ p-value	INL (131) R^2^ p-value	CNL (143) R^2^ p-value
FNL		0.045 0.60	-0.09 0.33
INL	0.045 0.60		0.23 0.008
**Food Label**			
Use of Label	-0.20 0.02	0.15 0.09	0.12 0.16
Look on ingredients	-0.11 0.23	0.52 0.000	0.23 0.006
Low fat	-0.14 0.12	0.32 0.000	0.04 0.62
Low calorie	-0.11 0.19	0.44 0.000	0.11 0.18
Serving size	0.01 0.91	0.30 0.000	0.09 0.3
Health Benefit	-0.12 0.16	0.34 0.000	0.08 0.32
Age (y)	-0.02 0.79	0.10 0.25	0.10 0.23
BMI (Kg/m^2^)	-010 0.24	0.03 0.71	-0.12 0.15
Diet Behavior			
Use of low calorie fat products	0.14 0.11	0.27 0.002	0.02 0.84
Use of low fat meat	0.002 0.98	0.28 0.002	0.08 0.34
Use of low fat milk	0.07 0.43	0.14 0.10	-0.03 0.73
Use of low fat cheese	-0.01 0.93	0.13 0.14	0.03 0.72
Use of frozen Yogurt	-0.003 0.97	0.12 0.18	0.08 0.34
Use of low calorie seasoning	-0.14 0.1	0.22 0.01	0.13 0.11
Use of fried Potatoes	-0.04 0.65	-0.17 0.056	-0.04 0.60
Frying veggi	0.15 0.09	-0.11 0.20	0.03 0.70
Adding cheese and mayonnaise	0.06 0.49	-0.11 0.23	-0.25 0.003
Use of Butter on breadand cake	-0.03 0.75	-0.03 0.73	-0.23 0.006
Avoid fat	0.07 0.4	0.25 0.003	0.15 0.06
Use of fried chicken	0.09 0.29	-0.22 0.01	-0.11 0.18
Remove chicken skin	0.09 0.28	0.32 0.000	0.05 0.57

FNL, functional nutrition literacy, INL, interactive nutrition literacy, CNL, critical nutrition literacy, R^2^, correlation coefficient

**Table 5:** Pearson correlation of study variables and measure of seeking nutrition Information

Variable	FNL R^2^ p-value	INL R^2^ p-value	CRL R^2^ p-value
efforts to obtain information	0.26 0.003	0.11 0.21	0.11 0.18
Not sure of information credibility	0.19 0.03	-0.03 0.77	-0.02 0.79
hard to understand nutrition information	0.58 0.000	-0.004 0.96	0.06 0.50
no enough information about nutrition in Arabic	0.28 0.001	0.1 0.27	0.12 0.17
takes a lot of time to search for information	0.33 0.000	0.11 0.23	0.06 0.46
**Use of source of nutrition information**			
Doctor, nurse, health worker	-0.15 0.08	0.17 0.05	0.11 0.19
Dietitian	-0.20 0.02	0.03 0.70	0.08 0.33
Family	-0.04 0.63	0.03 0.73	0.08 0.33
Friend	-0.08 0.35	0.02 0.84	-0.02 0.83
School Books	-0.17 0.05	0.02 0.84	-0.02 0.83
Newspapers and magazines	-0.12 0.15	-0.07 0.41	0.12 0.14
Internet	0.08 0.38	0.03 0.74	0.03 0.74
TV and Radio	-0.003 0.97	0.05 0.55	0.09 0.27
Government institution	0.02 0.87	0.002 0.98	0.11 0.19
International Organization	-0.31 0.000	-0.10 0.24	0.02 0.83

FNL, functional nutrition literacy, INL, interactive nutrition literacy, CNL, critical nutrition literacy, R^2^, correlation coefficient, TV, television

## Discussion

In this study, we describe three types of nutrition literacy in a group of Palestinians, primarily young people who study or work in health-related fields. We also found that nutrition label use, nutrition information seeking, and diet behavior are all significantly related to nutrition literacy.

People with higher FNL were less likely to engage in poor dietary habits. Furthermore, participants with a higher level of FNL appeared to trust nutrition information sources such as doctors, nurses, books, and the internet. Previous research has found a link between diet behavior and nutrition literacy [23, 24]. As a result, improved nutrition literacy may help to prevent chronic diseases [25].

In terms of FNL, our study group demonstrated a lack of knowledge regarding WHO guidelines for a healthy diet and the application of dietary guidelines to daily living. They demonstrated a strong understanding of nutrition knowledge and languages, which significantly correlated with a low-calorie and low-fat diet. However, this could be an overestimation of our study group FNL, because WHO healthy dietary guidelines include other aspects that were not included in this study, such as increased consumption of fruits and vegetables and decreased consumption of soft drinks, among other things [24, 26]. For example, the participant’s confidence in the definition of a healthy diet was high, but their knowledge of WHO guidelines on healthy nutrition was low, indicating that they may have overestimated their nutrition knowledge.

In terms of INL, participants were confident in sharing nutrition information and influencing peers and family, as well as health professionals, whereas the internet was not well received as a source of information. A study of women who used Facebook as a source of information on eating disorders found that it was associated with disorganized eating and a negative body image [27].

The study group is critical of the workplace, university, and workplace attitudes toward providing nutritious meals. Furthermore, the study group demonstrated a proclivity to influence and be influenced by others. They are more confident in what they believe to be a reliable source of information. Previous research found that when confronted with health issues such as diabetes, young students used websites such as Facebook, Twitter, and YouTube to obtain diet information [28].

Nutrition knowledge was linked to a lower intake of fat-rich foods and calories. A high-cholesterol, saturated fat and trans-fat diet are associated with an increase in serum low-density lipoprotein [29]. To reduce the risk of cardiovascular disease, the amount of saturated and trans fat in the diet should be reduced [30, 31]. Food selection is related to nutrition knowledge in the literature [32, 33], as some nutrition fundamental knowledge is related to diet change [34]. In a study of 231 students, those who got more than 35% of their total calories from fat had lower nutrition knowledge scores, and females had more nutrition knowledge than males.

Females had higher nutrition literacy than males. In line with previous research, nutrition knowledge in terms of nutrition recommendations, food nutrients, food choices, and diet-related diseases was higher in females than males, though total knowledge and nutrition recommendations were significantly higher in males [35]. Other studies show demographic differences in nutrition knowledge, with lower SES, unemployment, and less education having lower knowledge scores [36]. Nutrition knowledge is especially important for women because it helps to protect their children from malnutrition or future over-nutrition [37].

The Mediterranean diet is is popular in countries such as Palestine. Greater nutrition education was associated with higher adherence to the Mediterranean diet in a study of 127 students [38]. As a result, nutrition education to improve nutrition literacy among Palestinians may have an impact on the societal prevalence of chronic diseases.

## Study limitation

Due to limited resources, this study has some limitations, including the use of a convenient sample and a cross-sectional design. Because data collection took place during the corona lockdown, access to more participants was limited. Our sample consisted primarily of young students who had completed or were currently enrolled in a basic clinical nutrition class; thus, this sample does not represent the general Palestinian population, which may be lacking in nutrition knowledge, indicating the need for programs to improve nutrition literacy among Palestinians.

## Conclusion

In this study, we presented descriptive data on various types of nutrition literacy, which revealed that a young group of Palestinians, primarily university students, are interested in learning more about nutrition from credible sources, but find it difficult to apply a healthy diet in everyday life. CNL and INL were highly correlated. Nutrition literacy was found to be significantly related to label use, diet behavior, and credible sources of nutrition information. Efforts are needed to improve Palestinians’ nutrition knowledge. More research on nutrition literacy is required in various subgroups, including adolescents, middle-aged people, and the elderly. Efforts to improve nutrition literacy could have a significant impact on society in terms of disease prevention.

## Data Availability

The datasets generated and/or analyzed during the current study are not publicly available due [being kept confidential for future work] but are available from the corresponding author on reasonable request.
